# Editorial Comment: The clinical role of LASER for vulvar and vaginal treatments in gynecology and female urology: An ICS/ISSVD best practice consensus document

**DOI:** 10.1590/S1677-5538.IBJU.2020.03.08

**Published:** 2020-02-20

**Authors:** M Preti, P Vieira-Baptista, GA Digesu, CE Bretschneider, M Damaser, O Demirkesen, Cássio L. Z. Riccetto

**Affiliations:** 1 University of Torino Department of Obstetrics and Gynecology Torino Italy Department of Obstetrics and Gynecology, University of Torino, Torino, Italy; 2 Hospital Lusíadas Porto Porto Portugal Hospital Lusíadas Porto, Porto, Portugal; 3 Centro Hospitalar de São João Lower Genital Tract Unit Porto Portugal Lower Genital Tract Unit, Centro Hospitalar de São João, Porto, Portugal; 4 Imperial College Healthcare Department of Urogynaecology London UK Department of Urogynaecology, Imperial College Healthcare, London, UK; 5 Cleveland Clinic Obstetrics, Gynecology and Women's Health Institute Center for Urogynecology and Pelvic Reconstructive Surgery ClevelandOhio USA Center for Urogynecology and Pelvic Reconstructive Surgery, Obstetrics, Gynecology and Women's Health Institute, Cleveland Clinic, Cleveland, Ohio; 6 Cleveland Clinic Glickman Urological and Kidney Institute Department of Biomedical Engineering Lerner Research Institute ClevelandOhio USA Glickman Urological and Kidney Institute and Department of Biomedical Engineering Lerner Research Institute, Cleveland Clinic, Cleveland, Ohio; 7 Louis Stokes Cleveland VA Medical Center Advanced Platform Technology Center ClevelandOhio USA Advanced Platform Technology Center, Louis Stokes Cleveland VA Medical Center, Cleveland, Ohio; 8 Istanbul University Cerrahpaşa Faculty of Medicine Department of Urology Istanbul Turkey Faculty of Medicine, Department of Urology, Istanbul University Cerrahpaşa, Istanbul, Turkey; 1 Faculdade de Ciências Médicas da Universidade Estadual de Campinas – UNICAMP Divisão de Urologia Feminina CampinasSP Brasil Divisão de Urologia Feminina - Faculdade de Ciências Médicas da Universidade Estadual de Campinas – UNICAMP, Campinas, SP, Brasil

## COMMENT

The rationale for the use of LASER, radiofrequency and focused ultrasound in Female Urology is based on the application of concentrated and controlled amount of energy to the subepithelial vaginal tissues, with the objective of triggering an inflammatory response that leads to collagen deposition and angiogenesis. It is theorized that these phenomena would determine the recovery of normal vaginal physiology. Although theoretically consistent, the real effectiveness of LASER, radiofrequency and focused ultrasound has not yet been adequately proven. However, in recent years, there has been a large proliferation of their indications, both in the medical and physical therapy field, for various conditions.

The present review was published by a panel of experts and followed the ICS White Paper about Standard Operating Procedures. According to the authors, the majority of studies available so far have not been randomized, were composed of small series, had short follow-ups and lacked control groups. The authors also noted that until now, studies on LASERS have been funded by industry and have not been compared with conventional treatments, so that the level of evidence is low to allow any recommendation for clinical use. In fact, most references from the review were categorized as level of evidence C or D, regardless the type of clinical indication. According to the authors, although tissue alterations have been described in some publications, they mostly consist of reparative tissue alterations after a thermal injury such that it is difficult to establish a causal link between histological alterations and possible functional recovery.

Regarding urinary incontinence, six studies were included in the review, which comprised 19 to 205 patients without a control group, and response to treatment was assessed with short-term validated questionnaires (only one study referred to follow-up for 24 months).

Over the past 20 years, a myriad of treatments has been proposed for female urinary incontinence. Most of them were described as highly effective in initial publications, most of them with a short-term follow up. The advent of more developed scientific methods has shown that mid-urethral slings have proved to be reliable and safe long-term options for female incontinence. We expect that the use of laser and other energy sources in Female Urology diseases should soon be studied with the same quality standards.

## References

[1] 1. Salvatore S, Athanasious S, Yuen HTH, Karram M. LASER users' expert opinion in response to “The clinical role of LASER for vulvar and vaginal treatments in gynecology and female urology: An ICS/ISSVD best practice consensus document”. Neurourol Urodyn. 2019;38:2383-4. 10.1002/nau.24140 31432545

